# Investigating Synthesis of the MalS Malic Enzyme during Bacillus subtilis Spore Germination and Outgrowth and the Influence of Spore Maturation and Sporulation Conditions

**DOI:** 10.1128/mSphere.00464-20

**Published:** 2020-08-05

**Authors:** Bhagyashree Swarge, Chahida Nafid, Norbert Vischer, Gertjan Kramer, Peter Setlow, Stanley Brul

**Affiliations:** a Department of Molecular Biology and Microbial Food Safety, University of Amsterdam, Amsterdam, The Netherlands; b Department of Mass Spectrometry of Biomolecules, Swammerdam Institute for Life Sciences, University of Amsterdam, Amsterdam, The Netherlands; c Department of Molecular Biology and Biophysics, UConn Health, Farmington, Connecticut, USA; University of Iowa

**Keywords:** spore maturation, *B. subtilis*, MalS, protein synthesis, spore germination

## Abstract

Spore-forming bacteria of the orders *Bacillales* and *Clostridiales* play a major role in food spoilage and foodborne diseases. When environmental conditions become favorable, these spores can germinate as the germinant receptors located on the spore’s inner membrane are activated via germinant binding. This leads to the formation of vegetative cells via germination and subsequent outgrowth and potential deleterious effects on foods. The present report focuses on analysis of the synthesis of the MalS (malic enzyme) protein during Bacillus subtilis spore germination by investigating the dynamics of the presence and fluorescence level of a MalS-GFP (MalS-green fluorescent protein) fusion protein using time-lapse fluorescence microscopy. Our results show an initial increase in MalS-GFP fluorescence intensity within the first 15 min of germination, followed by a discernible drop and stabilization of the fluorescence throughout spore outgrowth as reported previously (L. Sinai, A. Rosenberg, Y. Smith, E. Segev, and S. Ben-Yehuda, Mol Cell 57:695–707, 2015, https://doi.org/10.1016/j.molcel.2014.12.019). However, in contrast to the earlier report, both Western blotting and SILAC (stable isotopic labeling of amino acids in cell culture) analysis showed there was no increase in MalS-GFP levels during the 15 min after the addition of germinants and that MalS synthesis did not begin until more than 90 min after germinant addition. Thus, the increase in MalS-GFP fluorescence early in germination is not due to new protein synthesis but is perhaps due to a change in the physical environment of the spore cores. Our findings also show that different sporulation conditions and spore maturation times affect expression of MalS-GFP and the germination behavior of the spores, albeit to a minor extent, but still result in no changes in MalS-GFP levels early in spore germination.

**IMPORTANCE** The spores formed by Bacillus subtilis remain in a quiescent state for extended periods due to their dormancy and resistance features. Dormancy is linked to a very low level of core water content and a phase-bright state of spores. The present report, focusing on proteins MalS and PdhD (pyruvate dehydrogenase subunit D) and complementary to our companion report published in this issue, aims to shed light on a major dilemma in the field, i.e., whether protein synthesis, in particular that of MalS, takes place in phase-bright spores. Clustered MalS-GFP in dormant spores diffuses throughout the spore as germination proceeds. However, fluorescence intensity measurements, supported by Western blot analysis and SILAC proteomics, confirm that there is no new MalS protein synthesis in bright-phase dormant spores.

## Abstract (precis)

The spores formed by Bacillus subtilis remain in a quiescent state for extended periods due to their dormancy and resistance features. Dormancy is linked to a very low level of core water content and a phase-bright state of spores. The present report, focusing on proteins MalS and PdhD (pyruvate dehydrogenase subunit D) and complementary to our companion report published in this issue, aims to shed light on a major dilemma in the field, i.e., whether protein synthesis, in particular that of MalS, takes place in phase-bright spores. Clustered MalS-GFP in dormant spores diffuses throughout the spore as germination proceeds. However, fluorescence intensity measurements, supported by Western blot analysis and SILAC proteomics, confirm that there is no new MalS protein synthesis in bright-phase dormant spores.

## INTRODUCTION

Sporulation and germination of genetically identical bacterial cells or spores have been extensively studied and shown to occur heterogeneously. This observation has challenged the food industries and the medical sector to extensively investigate both these processes to minimize microbiological risks by developing novel targeted strategies to eliminate spores. However, empirical studies on spores fail to reveal all details of the mechanistic basis of the spore’s resistance to the generally deployed bacterial elimination methods (1–3). Therefore, gaining detailed knowledge of sporulation and spore germination is of utmost importance. The resistance properties of spores mainly result from their layered structure and the chemical composition of those various layers (2, 4), while the germination receptor proteins (GRs), located in spores’ inner membranes (IM), facilitate spore germination (5, 6). In Bacillus subtilis spores, three GRs are well known—GerA, GerB, and GerK. Each of these proteins is composed of three subunits and can be activated by several types of germinants, including specific amino acids (4, 7). It is inferred that amino acids, such as alanine or valine, initially bind to the A and or B subunits of these receptors, thereby causing their activation (4, 8, 9). Once the GRs are activated, monovalent and divalent cations are released from the spores along with the spore core’s huge deposit (∼20% of core dry weight) of dipicolinic acid (DPA). Degradation of the peptidoglycan cortex then follows (7) and is a prerequisite for subsequent spore outgrowth. The occurrence of all these events leads to complete rehydration of the spore and restores its macromolecular synthesis and endogenous enzyme activity (6).

During spore outgrowth, complex sugars, carbohydrates, and organic acids can be used as carbon sources. In the case of B. subtilis, glucose and malate are generally the preferred carbon sources (10), with malate converted by malic enzymes (MalS, MaeA, MleA, and/or YtsJ) to pyruvate through oxidative decarboxylation (10) and then pyruvate oxidized to acetate. However, spores lack most tricarboxylic acid (TCA) cycle enzymes. Recently, Sinai et al. (11) investigated the synthesis of a MalS-GFP (MalS-green fluorescent protein) reporter fusion protein over time and suggested that MalS is one of the earliest proteins produced in germinating B. subtilis spores, as they observed a striking increase in GFP intensity in the first 5 min of spore germination. Remarkably, the fluorescence intensity of these spores increased even while they remained phase bright in phase-contrast microscopy, indicating that spore core hydration was not yet complete, thus lending support to their conclusion that protein synthesis is necessary during germination. In contrast, a subsequent study proved that protein synthesis is not essential for germination (12). Parallel to these observations, our proteomics studies (29) found that the levels of MalS in dormant spores are quite high and that there is no apparent change in its levels during germination. The studies mentioned above, however, differed with respect to the ages of the spores used in each study and in the media used for sporulation. It was reported previously that sporulation conditions and spore maturation times affect spore characteristics and resistance properties (13). Spores formed on solid rich media are generally more heat resistant and are characterized by a higher number of coat proteins involved in cross-linking (13). It was also shown previously that higher levels of spore coat protein cross-linking correlate with slight delayed germination times (12). Therefore, with respect to MalS synthesis and MalS-GFP expression during germination, the following research questions require attention. (i) Is MalS indeed synthesized in phase-bright spores soon after germinant addition? (ii) Do different sporulation conditions and spore maturation times affect MalS-GFP levels in B. subtilis spores?

To answer these questions, we have compared the germination characteristics of spores of B. subtilis strain PY79 and its AR71 (MalS-GFP) derivative prepared in liquid minimal medium and on solid rich medium. The germination of young (day 2) and older/mature (day 4) spores was triggered with a mixture of l-asparagine, d-glucose, d-fructose, and KCl (AGFK) plus l-alanine via the GerA/B/K germinant receptors. The time when germination started and the actual germination time, which is the time required for the spore’s transition from the phase-bright state to the phase-dark state, were then analyzed using time-lapse phase-contrast microscopy. MalS-GFP fluorescence in young and matured spores obtained from liquid and solid media was monitored during germination by fluorescence microscopy. In addition, synthesis of MalS-GFP during the initial stages of germination was followed by Western blot analysis, as was synthesis of MalS by SILAC (stable isotopic labeling of amino acids in cell culture) incorporation. We observed that the sporulation conditions and the length of the maturation period affected the germination of the spores. However, under no conditions was there any synthesis of MalS-GFP or MalS until long after spore germination was completed; thus, MalS synthesis, and, by extension, protein synthesis, does not take place in a phase-bright germinating spore.

(This research was conducted by B. Swarge in partial fulfillment of the requirements for a doctoral [Ph.D.] degree from the University of Amsterdam, The Netherlands [14]).

## RESULTS

### Dynamics of MalS and PdhD-IpHluorin fluorescence during germination and outgrowth.

**FIG 1:**
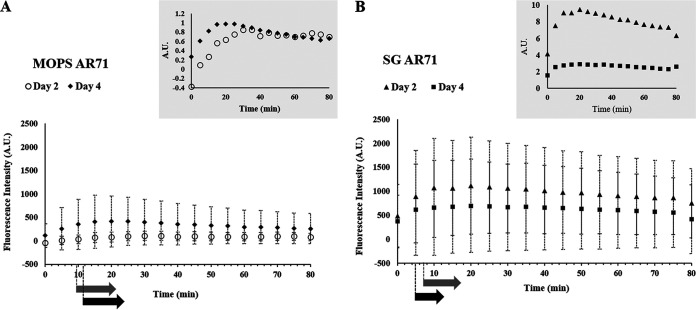
Real-time MalS-GFP fluorescence imaging during spore germination. The fluorescence intensities at germinating day 2 (*n* = 126) and day 4 (*n* = 425) for AR71 spores prepared in MOPS liquid medium (A) and at germinating day 2 (*n* = 118) and day 4 (*n* = 239) for spores prepared on 2× SG solid medium (B) were measured after initiation of germination. The fluorescence intensity of the wild-type spores was subtracted from the MalS-GFP fluorescence intensity to eliminate the background fluorescence of the spore itself. The subtracted values were 5% and 30% of the zero time value for day 2 and day 4 spores prepared on 2× SG medium, respectively, and were 79% and 48% of the zero time value for day 2 and day 4 spores prepared in MOPS medium, respectively. Also, the background values measured for these samples changed significantly during spore germination. The insets show the fluorescence intensity determined for an individual spore. The black and gray arrows at the bottom indicate the average germination times (see Table S1) for day 2 and day 4 spores, respectively, where the start and end positions of the arrows indicate the start and completion of germination, respectively. A.U., arbitrary units.

**FIG 2:**
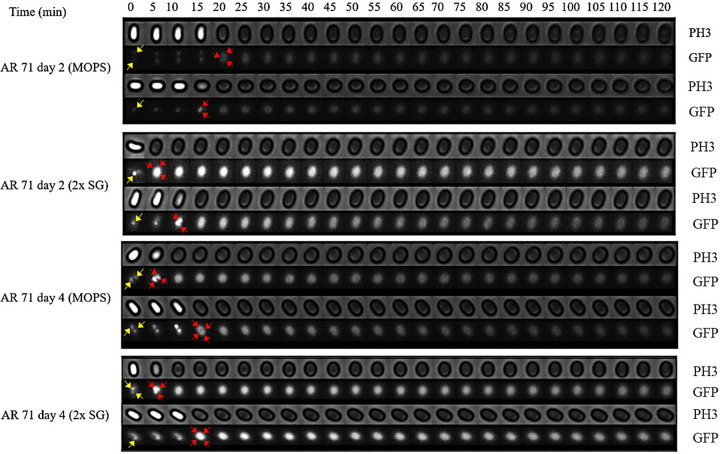
Phase-contrast (PH3) and fluorescence (GFP) images of germinating B. subtilis AR71 (MalS-GFP) young and mature spores prepared on solid and liquid media. Images of individual spores, showing GFP foci (yellow arrows), were captured every 5 min for 2 h to observe the phase-contrast transition (bright to dark) and changes in MalS-GFP fluorescence intensity. The diffuse GFP foci are shown by red arrows. As representatives of the data set, only two spores per sporulation condition are shown.

**FIG 3:**
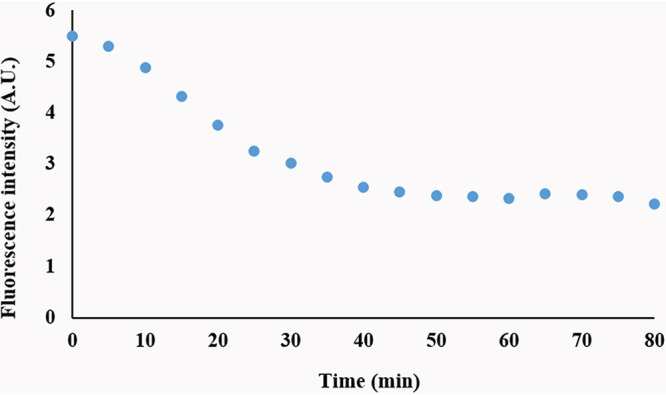
Changes in fluorescence intensity of young, MOPS medium-grown B. subtilis PdhD-IpHluorin spores during germination. The fluorescence intensity of spores from germinating day 2 (*n* = 87) PdhD-IpHluorin spores prepared in MOPS liquid medium was measured over 80 min after initiation of germination. The fluorescence intensity of the wild-type spores was subtracted from the PdhD-IpHluorin fluorescence intensity to eliminate the background fluorescence of the spore itself. The subtracted value was 2.3% of the zero time value.

**FIG 4:**
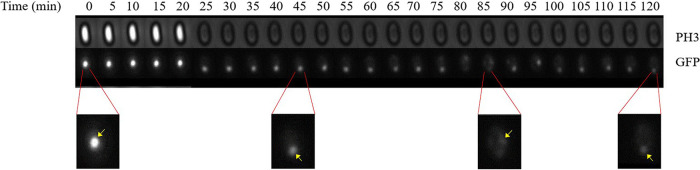
Fluorescence images of germinating young B. subtilis PdhD-IpHluorin spores prepared in MOPS medium. Shown is a collage of images of an individual germinating spore captured at different time points. The images were captured every 5 min using two different channels: PH3 (phase contrast) and GFP (IpHluorin). The PH3 channel visualizes the germination over time (phase bright to phase dark). The GFP channel visualizes changes in IpHluorin fluorescence intensity over time. The fluorescent foci are indicated by yellow arrows.

We initially assessed levels of MalS protein during spore germination and outgrowth by making use of MalS-GFP fluorescence, which was assessed by measurement of the fluorescence intensity of MalS-GFP for single spores throughout germination and outgrowth. Notably, spores prepared in different media exhibited different levels of MalS-GFP fluorescence, with levels higher in spores prepared in rich 2× Schaeffer’s medium with glucose (2× SG), and also differences in spores matured for 2 or 4 days (Fig. 1). As reported previously (11), populations of all 4 types of spores also showed significant increases in MalS-GFP fluorescence very early in spore germination, and this was also seen when the MalS-GFP fluorescence and phase-contrast image intensity of individual spores was monitored during germination (Fig. 1 insets; see also Fig. 2). As expected, the times for spore germination differed significantly between individual spores in the populations. More importantly, the times for the increases in MalS-GFP fluorescence following germinant addition often preceded the full change in the phase-contrast intensity (Fig. 2) and thus were often taking place before germination was complete. Notably, MalS-GFP was often but not always present in small foci in the dormant spores (yellow arrowheads in first images in Fig. 2), with 1 to 3 foci/spore seen in those spores that contained these foci (Table 1). These foci all appeared to coalesce to fill the spore core after the phase-bright-to-phase-dark transition (red arrowheads in Fig. 2) indicative of the completion of spore germination (Fig. 2). As spore outgrowth proceeded, the MalS-GFP intensities of the spores decreased slightly starting at 20 to 30 min and then remained relatively constant for the duration of the experiment (Fig. 1 and 2). In contrast to the behavior of MalS-GFP seen during spore germination, the fluorescence intensity of IpHluorin fused to another crucial metabolic protein, the d-subunit of pyruvate dehydrogenase (PDH), decreased in the first 30 min of germination and then remained constant during further measurements (Fig. 3). As noted above, young phase-bright dormant MalS-GFP spores, sporulated in either liquid or solid medium, showed only a small focus of GFP fluorescence, which became diffuse throughout the spore following completion of germination. Single fluorescent foci were also present in dormant young PdhD-IpHluorin spores made in minimal medium, but, in contrast to the MalS-GFP foci, the PdhD-IpHluorin foci persisted until well after germination was completed (Fig. 4).

**TABLE 1:** Heterogeneity in numbers of MalS-GFP foci in phase-bright dormant spores^*a*^

Spore sample (B. subtilis AR71)	No. of spores analyzed		% of phase-bright spores with 0 to 3 foci			
	R1	R2	0	1	2	3
MOPS						
D2	37	48	29	41	27	3
D4	71	52	24	38	34	4

2× SG						
D2	49		18	39	23	20
D4	69		67	13	15	6

a Numbers of MalS-GFP fluorescent foci measured in the dormant (time zero) spores prepared in MOPS and 2× SG medium are presented. For spores prepared in MOPS, the numbers represent averages of results from two biological replicates (R1 and R2). D2 (day 2) and D4 (day 4) represent the age of dormant spores.

10.1128/mSphere.00464-20.3 TABLE S1 Germination dynamics of young and mature spores of B. subtilis wild-type strain PY79 and mutant strain AR71 (MalS-GFP) prepared in liquid (MOPS) and solid (2× SG) media. *, germination time; §, cumulative percentage of spores that started germination at the indicated times. Download Table S1, PDF file, 0.1 MB. Copyright © 2020 Swarge et al. 2020 Swarge et al. This content is distributed under the terms of the Creative Commons Attribution 4.0 International license.

### Analysis of MalS-GFP levels and MalS synthesis during spore germination and outgrowth.

**FIG 5:**
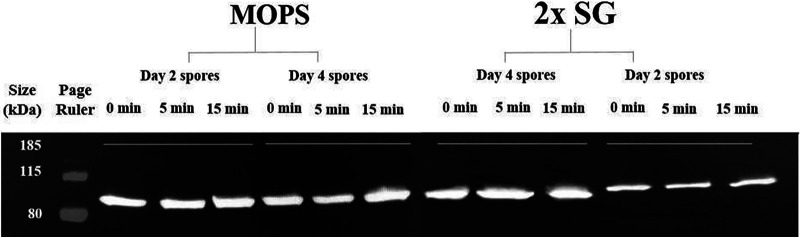
Western blot analysis of levels of MalS-GFP protein during spore germination. Dormant AR71 spores were germinated, protein was extracted from spores harvested before (*t* = 0) and after (*t* = 5, 15 min) germinant addition, spores were harvested and extracted, samples were run by the use of gel electrophoresis, and Western blot analysis carried out, all as described in Materials and Methods. For day 2 (young) spores prepared on 2× SG medium, 15 μg of protein was loaded on gels; for day 4 (old) spores, 30 μg of protein was loaded; and for day 2 (young) and day 4 (old) spores prepared in MOPS medium, 30-μg volumes of protein representative of all time points were loaded on gels. Spores from liquid medium were phase bright at *t* = 0 and 5 min and completed germination at 15 min. Spores obtained from solid medium completed germination at *t* = 5 min.

**FIG 6:**
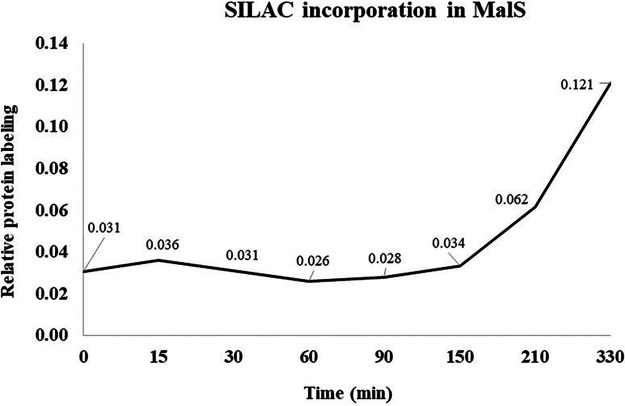
SILAC incorporation into MalS during spore germination and outgrowth. B. subtilis PY79 ^14^N-labeled spores (day 4) were germinated in MOPS minimal medium with l-lysine ^13^C_6_^15^N_2_ and l-arginine ^13^C_6_^15^N_4_ hydrochloride (Silantes; referred to here as SILAC) along with a mixture of AGFK and l-alanine as described previously (Swarge et al., submitted). Samples were taken at *t* = 0, 15, 30, 60, 90, 150, 210, and 330 min (*x* axis). Relative protein labeling (SILAC incorporation) results in terms of heavy/light ratios are shown on the *y* axis.

The increased MalS-GFP fluorescence seen very early in spore germination, in some cases before the phase-bright-to-phase-dark transition, suggested some MalS-GFP and MalS synthesis had taken place early in spore germination, and perhaps even in phase-bright spores. To directly assess whether there was any change in levels of MalS-GFP in the first 15 min after germinant addition, Western blot analyses was carried out on extracts from dormant and germinated young and mature AR71 spores prepared in liquid and on solid media and using an anti-GFP antiserum. This analysis showed clearly that the levels of the MalS-GFP fusion protein did not increase significantly within 15 min after the addition of the germinants, a time by which phase-contrast microscopy images showed that all spores had turned phase dark and thus that their germination was complete (Fig. 5 and data not shown). In addition, there was no SILAC incorporation in MalS protein until long after germination was completed in MOPS [3-(N-morpholino) propanesulfonic acid] medium (Fig. 6). Indeed, it was observed that incorporation of SILAC amino acids into MalS in this experiment did not begin until ≥90 min after the initiation of germination.

### Effect of sporulation conditions and maturation time on spore germination dynamics.

Given that the results obtained in our studies differed in some ways from previous work (11), we wished to be sure that the use of different methods of spore preparation had not drastically altered the germination process of the resultant spores. In general, the young and matured AR71 spores prepared on the solid medium required less time to complete germination (median germination time = 7.1 min and 8.2 min, respectively) than those prepared in the liquid medium (median germination time = 9.6 min and 8.7 min, respectively) (see Table S1 in the supplemental material; see also Fig. S1 and S2 in the supplemental material). The same trend was also observed in the matured WT spores (data not shown). Equally importantly, these slightly different germination parameters did not alter our fundamental finding of increased MalS-GFP fluorescence early in spore germination but no changes in MalS-GFP levels, as these were seen with the spores made under all four different conditions (Fig. 1, 2, and 5).

10.1128/mSphere.00464-20.1 FIG S1 Effect of sporulation media and spore maturation time on germination behavior. The data shown represent frequency distribution curves of B. subtilis wild-type strain PY79 and strain AR71 spores prepared in MOPS and 2× SG media. Germination time and germination start time are illustrated, and the germination conditions were as described in Materials and Methods. Data representing young spores (black line) are overlaid with data representing mature spores (red line). The significance of the data was examined using the Student *t* test. At the population level, for strain AR71 spores prepared in liquid medium, the relatively young spores were less prone to starting germination than the older spores (45% young spores versus 61% matured spores in 10 min) (Table S1A) and germination times did not differ significantly for these spore populations. The times required to start germination and the germination times also differed significantly for young and mature wild-type PY79 spores, and the young wild-type spores initiated germination later than matured spores (35% versus 44% in 10 min) (Table S1B). However, in sporulation on 2× SG solid medium, the young AR71 spores were more prone to starting germination than their mature counterparts (82% versus 71% in 10 min) (Table S1A). In contrast, the young PY79 spores were less prone to starting germination than the mature spores (67% versus 94% in 10 min) (Table S1B). The histogram is normalized based on the total spore count. Observations of two biological replicates were grouped and are analyzed as one data set. Download FIG S1, PDF file, 0.3 MB. Copyright © 2020 Swarge et al. 2020 Swarge et al. This content is distributed under the terms of the Creative Commons Attribution 4.0 International license.

10.1128/mSphere.00464-20.2 FIG S2 Effect of sporulation conditions on the germination behavior of young and mature spores of strains PY79 (WT) and AR71 (MalS-GFP). Conditions for germination and monitoring germination were as described in Materials and Methods. The histogram is normalized based on the total spore count. Observations from two biological replicates were grouped and analyzed as one data set. The significance of the data was determined using the Student *t* test. In general, with the exception of AR71 mature spores, the PY79 and AR71 spores prepared on the rich medium started germination earlier than those prepared in the minimal medium. Download FIG S2, PDF file, 0.4 MB. Copyright © 2020 Swarge et al. 2020 Swarge et al. This content is distributed under the terms of the Creative Commons Attribution 4.0 International license.

## DISCUSSION

Dormant spores of B. subtilis return to life through germination in sequential steps. During the first steps of revival, dormant spores release cations, small molecules, and Ca-dipicolinic acid (CaDPA) from the core accompanied by an increase in core water content of ∼30%. This triggers cortex hydrolysis, further CaDPA release, and full core hydration. Completion of germination results in a phase-dark spore which is ready to start macromolecular synthesis. Evidence obtained over decades of work on spore germination indicates that there is no significant metabolic activity and no protein synthesis in a spore until completion of germination (4–6, 15). However, recent reports have suggested that protein synthesis is essential during germination and that dormant spores are metabolically active (11). In the present work, we focused on the synthesis of the MalS protein and its GFP fusion, as this fusion protein was reported to be synthesized very early in spore germination and even in phase-bright spores (11).

Notably, the MalS-GFP fluorescence intensity from dormant spores made on rich solid medium was higher than that from spores prepared in a poorer liquid medium. Since the levels of the MalS-GFP protein were almost identical in mature dormant spores made in the two media, their different fluorescence intensities might reflect variations in the level of the mature GFP fluorophore in the spores made under different sporulation conditions, as suggested previously (16). Whether this is indeed the case might be clarified by using a MalS fusion to a superfolding GFP derivative. However, it is also possible that other factors are involved, such as differences in the precise conditions of the core environment in the spores made under different conditions. It was also notable that MalS-GFP was present in small foci in dormant spores, which might well affect the protein’s fluorescence by self-quenching.

The most striking aspect of the MalS-GFP fluorescence, however, was the increased fluorescence intensity seen when spores germinated, with this increase clearly taking place in spores that had not completed germination, all as reported previously (11). However, Western blot analysis of MalS-GFP levels during the first 15 min of spore germination, when almost all spores had completed germination, showed clearly that the increased MalS-GFP fluorescence was not due to new protein synthesis. This was further validated by the lack of any SILAC incorporation into MalS during this period and even out to at least 90 min after germinant addition. Thus, these results show that MalS is not synthesized early in spore germination, and the same results, as well as the fact that maturation times for a variety of GFPs range between 4 to 28 min (17), make the notion of *de novo* synthesis of MalS-GFP in spores followed by its proper folding within 5 min after germinant addition, as suggested previously by Sinai et al. (11), conceptually challenging. However, we do not know why use of relatively similar approaches have given such different results for levels of MalS and the analysis of MalS biosynthesis early in spore germination and outgrowth in two laboratories.

While the increase in MalS-GFP fluorescence very early in spore germination is not due to new protein synthesis, the reason for the increased fluorescence intensity is not clear. One possible explanation is that the increased fluorescence is due to GFP folding and fluorophore development, which might have been (i) slowed in the MalS-GFP fusion compared to that taking place in GFP alone and (ii) interrupted during spore development, when the developing spore’s core water content decreases markedly (18); also, as observed previously, proper folding of GFP is necessary for the chromophore formation (19). Alternatively, a dehydrated intermediate of GFP has been identified (20) which requires a single protonation and dehydration cycle to attain functional maturity. Perhaps this process takes place when there are significant proton movements early in germination as the spore core experiences rehydration, first by an increase in core water to 45% of wet weight when CaDPA is excreted in 1 to 2 min and then more slowly when the core water content rises to 80% of wet weight in ∼10 min as the cortex is hydrolyzed and the spore core expands. Expression of GFP alone in the spore core and examination of the GFP fluorescence that occurs when spores germinate might aid in determining which of these explanations is correct. In contrast to MalS-GFP fluorescence, PdhD-IpHlourin fluorescence did not increase early in germination but instead decreased slowly. Again, it might be informative to express IpHlourin alone in the spore core and examine its fluorescence during spore germination.

One surprising result was that MalS-GFP appeared to be present in one or more foci in the dormant spore core and that these foci transitioned to a more diffuse appearance throughout the germinated spore core. It has been shown that a pH of 6.5 promotes aggregation of at least enhanced GFP (21). Since the pH in the spore core is between 6.0 and 6.4 (17, 22), the observed clustering of MalS-GFP could thus represent GFP aggregation. The diffusion of these clusters throughout the spore core early in germination might then be due to the known rapid increase in core pH to ∼7.8 at that time, as well as to the initial increase of ∼30% in spore core water content paralleling CaDPA release in 1 to 2 min, followed by slower cortex hydrolysis and full core hydration. In contrast to MalS-GFP fluorescence, PdhD-IpHlourin fluorescence decreased in germination, although generally only after the phase-bright-to-phase-dark transition. PdhD-IpHluorin was also not present in foci in the dormant spore but transitioned to a small single focus as germination took place. However, the reason for the formation of the latter focus is not clear; perhaps PdhD associates with the other PDH subunits in one particular location in the germinated spore, although there is no information on this point. Beginning at ∼30 min after the addition of germinants, the MalS-GFP fluorescence intensity also began to decrease slightly. The reason(s) for the decreases in the fluorescence intensities of these two fusion proteins is not clear, but the decreases might have been due to degradation of the fusion proteins.

Since the germination process is affected by sporulation conditions (23) and spore maturation (12), the discrepancy between the earlier studies plus our current results on the one hand and the work of Sinai and colleagues on the other (11) could have stemmed from differences in the spore preparations used in the various studies. In general, we found that the MalS-GFP spores prepared on solid rich medium started germination earlier than spores prepared in the poorer liquid medium, as reported previously (23). The young spores prepared in liquid and solid media also exhibited shorter average germination times than the mature spores. These differences, while not great, could be due to differences in the (i) thicknesses of the spore coat layers and the protein cross-linking therein (13) and/or (ii) numbers of germination proteins in the spores, in particular, GRs, as suggested previously (8). Most importantly, spores prepared with the different media or matured for various times all exhibited (i) an increase in MalS-GFP fluorescence very early in spore germination, although the magnitudes of the increase differed, and (ii) no changes in MalS-GFP levels during the 15-min period when almost all spores had germinated completely.

Interestingly, the level of malic enzyme MalS is high in spores (Swarge et al., submitted) whereas two other malic enzymes, YtsJ and YqkJ, are also present in both spores and growing cells. A recent study suggested that l-malate stores in spores are used as an energy source very early in germination, and MalS was reported to be one of the earliest proteins synthesized in spore germination (11). Since malate and glucose are preferred carbon sources for B. subtilis (10), the previous observations suggested that high levels of MalS could be important for spores during germination for utilization of each spore’s store of l-malate. However, older work as well as a very recent study revealed that levels of l-malate in spores are extremely low, casting significant doubt on its proposed role as an energy reserve in dormant spores (24, 25). However, the enzyme may well be very important in catabolizing the large amount of malate that accumulates in germinating spores, but only more slowly than ATP accumulation (25). The precise source of this accumulated malate is not clear, but the accumulation may a consequence of the breakdown of amino acids generated by degradation of spore protein. An in-depth study focusing on the details of malate metabolism in spores during germination and outgrowth is certainly needed.

Overall, the most important conclusion from this work is that whereas analysis of MalS-GFP fluorescence intensities confirmed the dramatic increase in MalS-GFP fluorescence early in spore germination reported previously (11), this increase was not accompanied by any changes in MalS-GFP protein levels or any in MalS synthesis at that time. Thus, synthesis of these proteins, and indeed perhaps any proteins, as shown recently (Swarge et al., submitted), is not important in B. subtilis spore germination.

## MATERIALS AND METHODS

### Bacterial strains and sporulation conditions.

B. subtilis wild-type strain PY79 (WT) and mutant strain AR71 with a PY79 background (MalS-GFP; spectinomycin resistant [Spc^r^]; obtained from Sinai et al. [11]) were used in this study. PdhD (pyruvate dehydrogenase subunit D) was also reported to be newly synthesized at an early stage in spore outgrowth (11). Therefore, as an independent control for the fluorescence measurements of MalS-GFP, we used a B. subtilis PY79 strain (erythromycin resistant [Erm^r^]) harboring a PdhD-IpHluorin (PC) fusion protein prepared in our laboratory, with the IpHluorin a GFP derivative (22). The two different sporulation media used in this study were defined liquid minimal medium buffered to pH 7.5 with 80 mM 3-(N-morpholino) propanesulfonic acid (MOPS) and rich 2× Schaeffer’s medium with glucose (2× SG) agar solid medium as described previously (12, 26). Bacterial cells were routinely cultured on a tryptic soy agar (TSA) plate and incubated overnight at 37°C. A single colony was inoculated in tryptic soy broth (TSB) medium and incubated at 37°C at 200 rpm until the exponential-growth phase was reached (optical density at 600 nm [OD_600_] of ∼0.3 to 0.4). A serial dilution series of the culture in 5 ml of MOPS liquid medium or 2× SG liquid medium was then generated and incubated overnight at 37°C with shaking at 200 rpm. A dilution containing exponentially growing cells was selected, and the bacterial culture was further inoculated in 20 ml MOPS liquid medium or 2× SG liquid medium. For sporulation, cells cultured in MOPS liquid medium were inoculated into 300 ml MOPS liquid medium and cultured at 37°C with shaking at 200 rpm, while cells cultured in 2× SG liquid medium were spread on 10 plates containing 2× SG agar and incubated at 37°C.

### Spore harvesting.

Spores cultured in MOPS liquid medium and on 2× SG solid medium were harvested after 48 and 96 h (13). Spore samples were extensively washed with prechilled Milli-Q water at 4°C. Subsequently, Tween 20 (0.01% [vol/vol]) was added to the spores in Milli-Q water to kill vegetative cells and to improve purification. The harvested spores were examined under a microscope using a hemocytometer, and the spore yield was estimated. Remaining vegetative cells and/or germinated spores (phase dark) were removed using the Histodenz gradient centrifugation method described previously by Abhyankar et al. (13). The purified harvested spores contained more than 99% phase-bright spores and were stored at −80°C until use.

### Germination and fluorescence measurements.

For time-lapse germination experiments, spores maintained at an OD of ∼1 were subjected to heat activation at 70°C for 30 min at 100 rpm and placed on ice for 10 min. Immediately after incubation on ice, the spores were transferred to an agarose pad containing 2% (wt/vol) agarose mixed with a double-strength concentration of MOPS medium and germinants (AGFK [10 mM l-asparagine, 10 mM d-glucose, 1 mM d-fructose, 1 mM KCl] and 10 mM l-alanine) in a 1:1 ratio. The technique of the setup of the microscope slide for time-lapse microscopy imaging was described previously by Pandey et al. (27). Time-lapse microscopy imaging was performed at 37°C for 2 h using a temperature-controlled incubation system. A Nikon Eclipse Ti-E inverted wide-field fluorescence time-lapse microscope equipped with an Apo total internal reflection fluorescence (TIRF) 100× H/1.49 lens objective and a Hamamatsu ORCA-AG cooled charge-coupled-device (CCD) camera with an integrated perfect focus system were used. Phase-contrast images were taken with an acquisition time of 100 ms. Fluorescence images were taken with a GFP filter and an IpHluorin filter with an acquisition time of 250 ms and at 50% power. Phase-contrast and GFP fluorescence time-lapse series were recorded at a sample frequency of 4 to 7 frames per 5 min, with about 100 spores examined per measurement. One replicate for the control PdhD-IpHluorin strain was also performed. Spore germination times and fluorescence intensity were both analyzed using SporeTrackerX (28) (https://sils.fnwi.uva.nl/bcb/objectj/examples/sporetrackerx/MD/sporetrackerx.html), a plugin for ObjectJ (https://sils.fnwi.uva.nl/bcb/objectj/). The time required for initiating the phase-bright-to-phase-dark transition after addition of germinants (here referred to as the start of germination) and the time required to complete the phase transition (here referred to as germination time) were analyzed. For GFP measurements, the fluorescence intensities of spores of a wild-type strain lacking the *gfp* gene (which differed between the spores prepared in MOPS and those prepared in 2× SG media) were subtracted from the net average fluorescence intensity. All statistical analysis was performed in SigmaPlot 13.0 (Systat Software Inc.). To analyze results of live-imaging data from two independent replicates, the Student *t* test was performed and *P* values were determined.

### Western blot analysis.

Spores of B. subtilis strain AR71 (MalS-GFP), prepared on solid and liquid media, were heat activated at 70°C for 30 min. Germination was triggered using the l-alanine/AGFK mixture, and spores were harvested before (*t* = 0 min) and after (*t* = 5, 15 min) the addition of the germinants. To stop further protein synthesis during sample processing, chloramphenicol (100 μg/ml) and methanol (20% [mass/vol]) were added to the samples. The spores were mixed in phosphate-buffered saline (PBS; 10 mM Na_2_HPO_4,_ 1.8 mM KH_2_PO_4,_ pH 7.5) containing protease inhibitor cocktail (cOmplete, Mini, EDTA-free protease inhibitor cocktail; Roche) and broken using a Precellys 24 homogenizer (Bertin Technologies, Aix-en-Provence, France; speed, 6.5, 40 s, 7 cycles). The protein concentration of each sample was measured using a Bradford assay kit (Bio-Rad, Utrecht). For every time point, equal concentrations of proteins were incubated at 100°C for 10 min with Laemmli sample buffer. The proteins were separated on a 10% Bis-Tris SDS gel and subjected to electroblotting overnight onto a polyvinylidene difluoride (PVDF) transfer membrane. A PageRuler prestained protein ladder (10 to 180 kDa) was used as a reference ladder. For immunoblot analysis of GFP fusion proteins, the membranes were blocked for 1 h at room temperature with a mixture containing 0.05% Tween 20, 5% skim milk, and 1× Tris-buffered saline (TBS) (pH 7.4). Blots were then incubated for 1 h at room temperature with polyclonal rabbit anti-GFP antibodies (1:10,000 in a mixture containing 0.05% Tween 20, 5% skim milk, and 1× TBS [pH 7.4]). Subsequently, the membranes were incubated overnight at 4°C with peroxidase conjugated anti-rabbit secondary antibody (1:10,000 in a mixture containing 0.05% Tween 20, 5% skim milk, and 1× TBS [pH 7.4]). A SuperSignal West Femto maximum sensitivity substrate kit was used for final detection of the protein on the blot.

### SILAC incorporation to assess MalS synthesis and proteomic analysis.

The SILAC incorporation approach (Swarge et al., submitted) was used for experimental tracing of the synthesis of the MalS protein during spore germination and outgrowth. Wild-type mature B. subtilis ^14^N-labeled spores (4 days old) were germinated at 37°C in MOPS minimal medium with l-lysine ^13^C_6_^15^N_2_ (Thermo Fisher Scientific, USA) (365 mg/liter) and l-arginine ^13^C_6_^15^N_4_ hydrochloride (Silantes; here referred to as SILAC) (Thermo Fisher Scientific, USA) (210 mg/liter) along with the mixture of AGFK and l-alanine as described above. Samples (20 ml) were taken at *t* = 0, 15, 30, 60, 90, 150, 210, and 330 min, and spores were harvested and processed using the previously published “one-pot” method (26).

The samples were further analyzed by liquid chromatography-tandem mass spectrometry (LC-MS/MS) on a timsTOF Pro (trapped ion mobility spectrometry coupled with quadrupole time of flight Pro) mass spectrometer (Bruker Daltonics, Bremen, Germany) equipped with an Ultimate 3000 RSLCnano ultra-high-performance liquid chromatography (UHPLC) system (Thermo Scientific). A 200-ng volume of a tryptic digest cleaned on a TT2 TopTips (Glygen) column (according to the manufacturer’s instructions) was injected into an Aurora C_18_ column (Ionopticks) (25-cm length by 75-μm inner diameter, 1.6-μm particle size). The peptides were eluted from the column by applying a gradient from 0.1% formic acid–3% acetonitrile (ACN) to 0.1% formic acid–85% ACN (flow rate, 400 nl/min) in 140 min. For the acquisition cycle, 10 PASEF (parallel accumulation serial fragmentation) MS/MS scans were acquired with a total cycle time of 1.16 s. MS and MS/MS spectra were recorded from 100 to 1,700 *m*/*z*, and precursor ions for PASEF scans were selected in real time by the precursor selection algorithm. A polygon filter was applied to the *m*/*z* and ion mobility plane to select the features most likely representing peptide precursors. For all experiments, the quadrupole isolation width was set to 2 Th for *m*/*z* values of <700 and 3 Th for *m*/*z* values of >700. Collision energy was ramped from 20 to 59 eV over the TIMS scan range. Data were processed with the MASCOT DISTILLER program (version 2.4.3.1, 64 bits) and MDRO 2.4.3.02 (Matrix Science, London, United Kingdom), including the Search toolbox against the B. subtilis 168 ORF translation database. Peaks were fitted to a simulated isotope distribution with a correlation threshold of 0.7, with a minimum signal-to-noise ratio of 2. The MASCOT search parameters were as follows: enzyme, trypsin; allowance of two missed cleavages; fixed modification, carbamidomethylation of cysteine; variable modifications, oxidation of methionine and deamidation of asparagine and glutamine; quantification method, SILAC K + 8 *R* + 10; peptide mass tolerance and peptide fragment mass tolerance, 50 ppm. A MASCOT MudPIT peptide identification threshold score of 20 and a FDR (false-discovery rate) value of 2% were set to export the reports. Using the quantification toolbox, the quantification of the incorporation of SILAC relative to the corresponding ^14^N peptides was determined as a ^SILAC^/^14^N ratio using Simpson’s integration of the peptide MS chromatographic profiles for all detected charge states. The quantification parameters were as follows: correlation threshold for isotopic distribution fit, 0.80; XIC threshold, 0.1; all charge states on; max XIC width, 120 s; elution time shift for heavy and light peptides, 10 s. All isotope ratios were manually validated by inspecting the MS spectral data. The protein isotopic ratios were then calculated as the average over the corresponding peptide ratios.

### Data accessibility.

The raw proteomics data from all the approaches have been deposited in the PRIDE repository with the data set identification number PXD018345.
