# Supplementary material for: Investigating Synthesis of the MalS Malic Enzyme during Bacillus subtilis Spore Germination and Outgrowth and the Influence of Spore Maturation and Sporulation Conditions
Source: mSphere. 2020 Aug 5;5(4):e00464-20. doi: 10.1128/mSphere.00464-20 (PMC7407067; doi:10.1128/mSphere.00464-20)
Supplement: TABLE S1 [file mSphere.00464-20-st001.pdf]

Supplementary Table

Table S1. Germination dynamics of young and mature spores of *B. subtilis* wild type strain PY79 and mutant strain AR71 (MalS-GFP) prepared in liquid (MOPS) and solid (2x SG) media.

	Day 2					Day 4				
**Medium of sporulation**	**Counts (n)**	**Avg. GT* (min)**	**Median GT (min)**	**Germination start time (min)**	**% of spores starting germination§**	**Counts (n)**	**Avg. GT (min)**	**Median GT (min)**	**Germination start time (min)**	**% of spores starting germination**
**(A) *B. subtilis* AR71 (MalS-GFP)**										
**MOPS (liquid)**	131	12.1±0.64	9.6	5	28	421	12.78± 0.7	8.6	5	34
				10	45				10	61
				15	73				15	83
**2x SG (solid)**	141	7.2± 0.28	7.1	5	66	237	10.0± 0.53	8.2	5	55
				10	82				10	71
				15	94				15	84
**(B) *B. subtilis* PY79 (WT)**										
**MOPS (liquid)**	199	9.6± 0.3	9.2	5	21	361	17.0±1.0	9.2	5	20
				10	35				10	44
				15	55				15	69
	294	11.5±0.63	8.7	5	48	278	8.5± 0.46	7.7	5	75

**2x SG (solid)**	10	67	10	94
	15	85	15	98
