# Supplementary figures and images for: Investigating Synthesis of the MalS Malic Enzyme during Bacillus subtilis Spore Germination and Outgrowth and the Influence of Spore Maturation and Sporulation Conditions
Source: mSphere. 2020 Aug 5;5(4):e00464-20. doi: 10.1128/mSphere.00464-20 (PMC7407067; doi:10.1128/mSphere.00464-20)

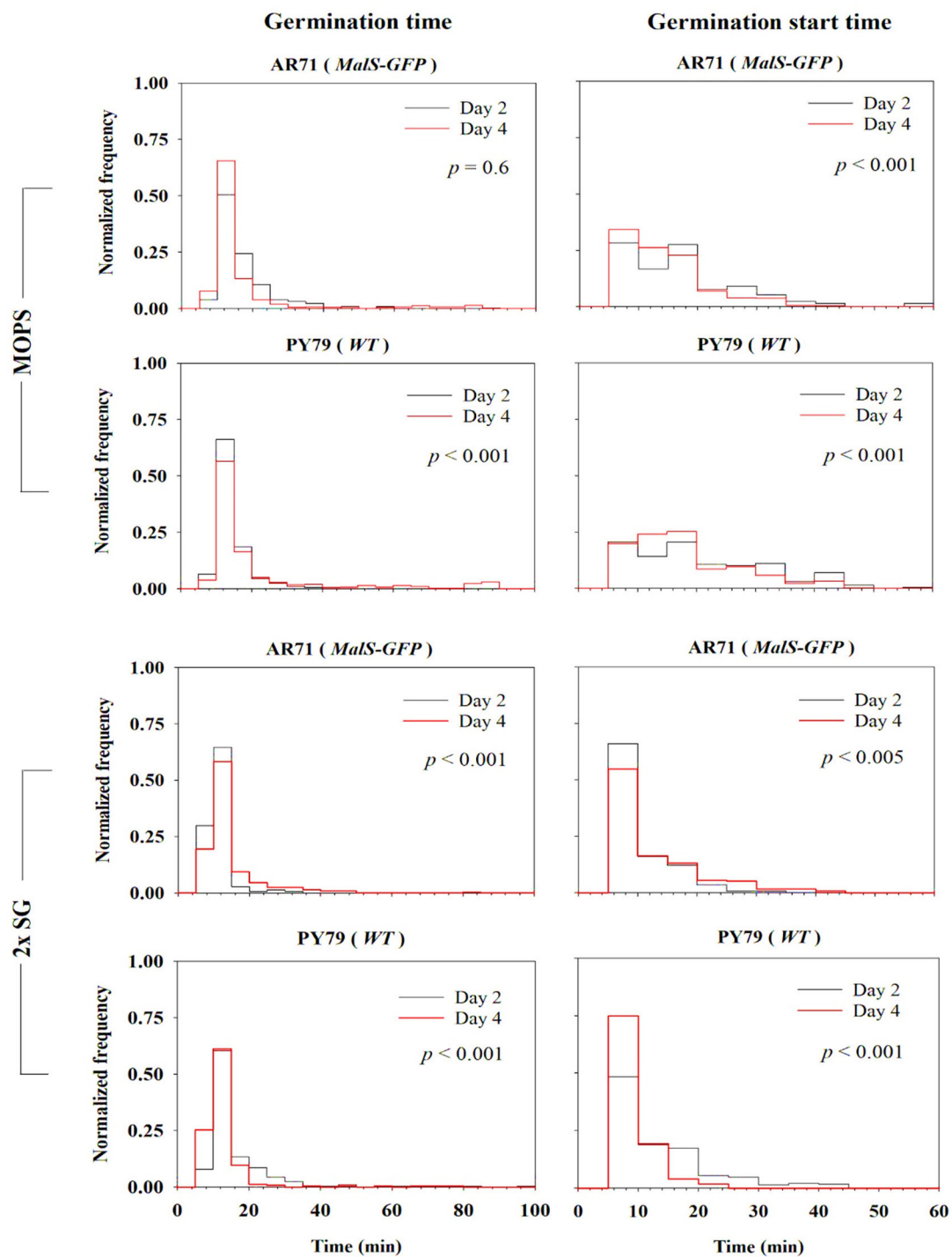

**Fig. S1. Effect of sporulation media and spore maturation time on germination behavior.**

Supplement: FIG S1 [file mSphere.00464-20-sf001.pdf]
