# Supplementary material for: Investigating Synthesis of the MalS Malic Enzyme during Bacillus subtilis Spore Germination and Outgrowth and the Influence of Spore Maturation and Sporulation Conditions
Source: mSphere. 2020 Aug 5;5(4):e00464-20. doi: 10.1128/mSphere.00464-20 (PMC7407067; doi:10.1128/mSphere.00464-20)
Supplement: FIG S2 [file mSphere.00464-20-sf002.pdf]

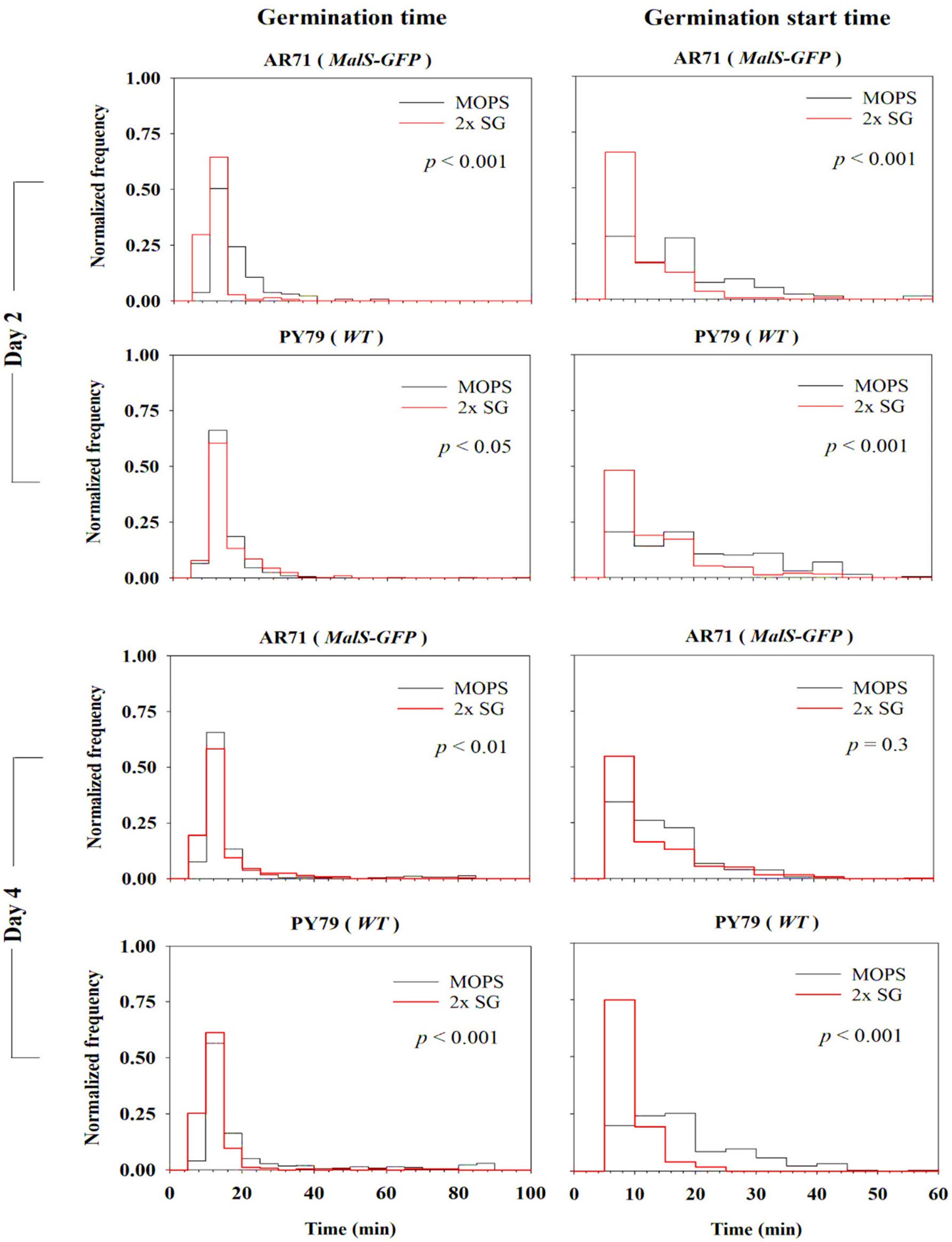

Fig. S2. Effect of sporulation conditions on the germination behavior of young and mature spores of strains PY79 (WT) and AR71 (MalS-GFP).
